# Postoperative Quality Assessment Score Can Select Patients with High Risk for Locoregional Recurrence in Colon Cancer

**DOI:** 10.3390/diagnostics12020363

**Published:** 2022-02-01

**Authors:** Cristian Livadaru, Mihaela Moscalu, Florina Adriana Ghitun, Alexandra Ramona Huluta, Cristina Terinte, Dan Ferariu, Sorinel Lunca, Gabriel Mihail Dimofte

**Affiliations:** 1 Surgical Department, Grigore T. Popa University of Medicine and Pharmacy, 700115 Iasi, Romania; cristian.livadaru@gmail.com (C.L.); sdlunca@yahoo.com (S.L.); gabriel.dimofte@umfiasi.ro (G.M.D.); 2 Radiology and Medical Imaging Department, St. Spiridon Emergency County Clinical Hospital, 700111 Iasi, Romania; 3 Department of Preventive Medicine and Interdisciplinarity, Grigore T. Popa University of Medicine and Pharmacy, 700115 Iasi, Romania; 4 G. Georgescu Institute of Cardiovascular Diseases, 700503 Iasi, Romania; flori.ghitun@gmail.com; 5 2nd Clinic of Surgical Oncology, Regional Oncology Institute, 700483 Iasi, Romania; alexandrahuluta90@yahoo.it; 6 Department of Pathology, Regional Oncology Institute, 700483 Iasi, Romania; cterinte@gmail.com (C.T.); d_ferariu@yahoo.com (D.F.)

**Keywords:** complete mesocolic excision, locoregional recurrence prediction score, surgical quality evaluation, postoperative CT, arterial stump measurement, colon cancer

## Abstract

Background: Monitoring surgical quality has been shown to reduce locoregional recurrence (LRR). We previously showed that the arterial stump length (ASL) after complete mesocolic excision (CME) is a reproducible quality instrument and correlates with the lymph-node (LN) yield. We hypothesized that generating an LRR prediction score by integrating the ASL would predict the risk of LRR after suboptimal surgery. Methods: 502 patients with curative resections for stage I–III colon cancer were divided in two groups (CME vs. non-CME) and compared in terms of surgical data, ASL-derived parameters, pathological parameters, LRR and LRR-free survival. A prediction score was generated to stratify patients at high risk for LRR. Results: The ASL showed significantly higher values (50.77 mm ± 28.5 mm) with LRR vs. (45.59 mm ± 28.1 mm) without LRR (*p* < 0.001). Kaplan–Meier survival analysis showed a significant increase in LRR-free survival at 5.58 years when CME was performed (Group A: 81%), in contrast to non-CME surgery (Group B: 67.2%). Conclusions: The prediction score placed 76.6% of patients with LRR in the high-risk category, with a strong predictive value. Patients with long vascular stumps and positive nodes could benefit from second surgery to complete the mesocolic excision.

## 1. Introduction

The principles of complete mesocolic excision (CME) for colon cancer (CC), as reinvigorated by Hohenberger [1], generated a decrease in local recurrence rate (LRR) to 3.3%. Similarly, Bertelsen [2] compared CME with conventional colectomy and showed a 4-year disease-free survival (referred to as DFS) of 85.8% with CME compared to 73.4% (non-CME) and a decrease in 5-year local recurrence, from 6.5% (non-CME), to 3.6% (CME), showing that the mesocolic resection technique is an independent prognostic factor in all operable patients.

CC surgery, however, is still a field of non-standardized colectomies, with proven variations amongst clinicians, hospitals, and countries. Complete mesocolic excision is not yet adopted as a gold standard recommendation to the same extent as total mesorectal excision is for rectal cancer. Some professional and national societies such as the American Japanese, Norwegian and French colon cancer societies recommend CME but with variations regarding the lymphadenectomy level [3,4,5,6,7,8]. The concept of CME with central vascular ligation (CVL) provides an elegant anatomical standardization, but it is still not used universally, with only tertiary centers and colorectal cancer specialists performing CME with consistent precision [2,9,10,11]. Moreover, surgical centers and countries with an interest in adopting CME with standardized systematic D_2_ or D_3_ lymphadenectomy have shown the benefits of a good surgical quality care program in reducing locoregional recurrence (LRR) [12,13,14,15].

Paralleling Heald’s concept of the mesorectal fascia, CME has emerged as an anatomical plane for an ontogenetic compartment surgical plane, using sharp dissection in the mesocolic avascular interface [16,17]. Few papers have discussed the issue of local and regional recurrences after oncologic resections for CC, while the status of circumferential radial margin after total mesorectal excision is a prominent topic [18,19,20,21]. The prognosis of rectal cancer has seen the most radical increase due to the improvement of the surgical technique, objectified by the decrease in LRR and sustained by quality evaluation programs, which has led many groups [1,2,22] to translate this technique of sharp dissection into CC surgery.

The literature on patients with LRR after curative resection for CC is scarce, and surgical details, incidence, treatment, or follow-up have not been thoroughly reported to the same extent as systemic oncologic outcomes [13,23]. The current European Society of Medical Oncology (commonly referred to as the ESMO) guidelines recommend adjuvant chemotherapy in cases with high-risk factors of recurrence for stage II CC, but do not discriminate specifically between local and systemic recurrences [24]. A validated Swedish registry of CC gives a very accurate view on the LRR rate and serves as proof of a good quality control program for CC surgery. Systematic reviews on LRR in CC suggest that local recurrence is rarely an outcome in CC trials, with most reported parameters being DFS and overall survival (OS) [11,12]. This registry had extensive follow-up of all the LRRs, and some of the risk factors discovered were the length of the vascular pedicle, distal ligature, or no reported ligature [12]. Generally, LRRs may be multifactorial, but the incidence should be minimal after oncological CME with CVL. Thus, local recurrence after resectable CC tends to be primarily a surgical quality problem with a suboptimal surgical specimen [2,11].

The optimum level of ligation in CME remains debatable, as several studies advocate that no further benefit can be derived after a central D_3_ vascular ligation [25,26]; other studies argue that CME with CVL produces a higher quality surgical specimen, with increased number of lymph nodes (LNs) and a resection within embryological anatomical planes [1,11,27,28,29,30,31]. 

Currently, the only method to evaluate surgical quality is through pathologically derived parameters, such as the number of harvested LNs, the number of positive LNs, and the LN index, all of which are quantitative indicators of surgical quality and represent validated prognostic markers of locoregional and systemic recurrences [24,32,33,34]. However, LN count can vary depending on pathological examination techniques and tumor factors [24,35]. The residual vascular pedicle length cannot be appreciated pathologically, while surgical protocols seldom describe the level of ligation, and if they do so, they only provide a qualitative description and not a quantitative input [13,24,36]. A predictable instrument of quality control was presented in our previous studies—the arterial stump length (ASL), which correlates with the number of harvested LNs. We believe that the actual quantitative length of the arterial stump, rather than the unspecific qualitative description of vascular ligation (D_2_/high/central/D_3_), could be a better quality marker and may be included in a score of LRR prediction, alongside the established risk factors [9,28].

Several studies flagged the vascular ligation level as a risk factor for LRR [6,10,28,36]. Due to the insertion of the mesocolon along the root of the mesenteric arteries, it would be logical to presume that a long arterial stump would be inherently associated with residual mesocolic tissue around it [37], thus posing a higher risk for recurrence, as demonstrated by West et al. [29] when they stratified survival according to mesenteric integrity. At the same time, Kaye [38] correlated longer stumps with a more frequent perforation of the mesocolic fascia using dedicated pathology examination protocols. Hence, a surgical gesture that leaves behind a long residual arterial stump represents a suboptimal specimen both in terms of incomplete lymphadenectomy and incomplete mesocolic excision. Furthermore, postoperative computer tomography (CT) studies evaluating the ASL unanimously showed that the D_2_ ligation standard is frequently not reached, suggesting the presence of residual mesenteric tissue and LNs [12,36,37,38,39]. The work of Sjövall [23] showed that once an LRR occurs, the only curative treatment would be the surgical re-intervention for completing the resection of the tumor with the residual mesocolon. The authors further demonstrated a dismal survival if re-surgery had not been performed. 

To this end, a score analysis was employed based on retrospective cohorts of patients with and without LRR after surgical treatment, using classic and modern multivariate analysis risk predictors in association with the quantitative CT measurements of ASL and derived ASL angiometry parameters to mathematically predict the importance of the ligation height on the overall LRR risk. Hypothetically, a patient with multiple negative prognostic factors would benefit from a second surgical intervention to complete the mesocolic excision before LRR occurs.

Our purpose was to generate a score for selecting patients at high risk of LRR after CC surgical treatment. Identification of such a subgroup may be used to support an indication for salvage surgical procedures for cases with suboptimal primary surgery with long arterial stumps, aiming to prevent LRR and unfavorable oncological prognosis. The score validation would be verified by applying it to the cohorts of patients with and without LRR after surgical treatment.

## 2. Materials and Methods

### 2.1. Design and Setting

This study was a retrospective analysis conducted on a prospectively maintained database of patients treated at a tertiary hospital—Regional Oncology Institute (IRO) Iasi, Romania.

The study cohort was divided into two groups of patients based on the surgical technique:

Group A included patients who were operated on at the IRO by 10 different surgeons. Colectomies were included consecutively over a 90-month period (March 2012 through September 2019). Colectomies from this group had been performed in compliance with the CME technique, with adjustments to the high vascular ligation level:

- high ligation of the ileocolic artery (ICA) adjacent to the lateral wall of the superior mesenteric vein (commonly referred to as SMV) in right colectomies (i.e., D_2_ lymphadenectomy);
- high ligation of the inferior mesenteric artery (IMA), at approximately 10 mm from the aorta in left colectomies;
- low ligation of the IMA in segmental colectomies for sigmoid cancer, just below the emergence of the left colic artery (LCA), but with further dissection around the IMA trunk up to its origin from the aorta (i.e., D_3_ lymphadenectomy).

Group B comprised patients operated on in other surgical units (general departmental hospitals), who had an oncologic registration in our institutional database in the same period of time. These patients were under oncologic treatment and surveillance in our institution. Operations in this group were non-CME, radical colectomies with curative intent. 

### 2.2. Patients and Selection Criteria

The study was approved by the Institutional Ethics Committee of the IRO, and patients had given consent for inclusion in the research database.

The study inclusion criteria were patients with CC stages I–III, according to the 8th edition of Union for International Cancer Control (commonly known as UICC), without preoperative chemotherapy, who underwent surgery with curative intent, with accessible good quality postoperative contrast-enhanced CT.

The exclusion criteria were stage IV CC (UICC 8th edition), palliative colectomies, transverse CC, perforated CC, age under 18 years, low quality CT examination or with large reconstruction interval (>5 mm), unavailable or incomplete pathological results, R1 resections, and pT4b stage.

In accordance with ESMO guidelines, all stage III patients and high-risk T3N0 patients positive for microsatellite instability were given adjuvant chemotherapy [24].

### 2.3. Data Collection and Database Architecture

The statistical indexes of the entire electronic database of the IRO were correlated with the disease codes for “colon cancer” as allocated by our national diagnosis related groups. Thus, an output of 3600 unique consecutive patients was generated and used as an instrument for systematic identification of all the patients eligible for the study. Hardcopy oncologic registries of the IRO Department of Oncology were used to retrieve essential medical information for patients in Group B. The CT images were accessed from the IRO Patient Archiving and Communication System (i.e., PACS) of the hospital’s intra-network.

**Figure 1:**
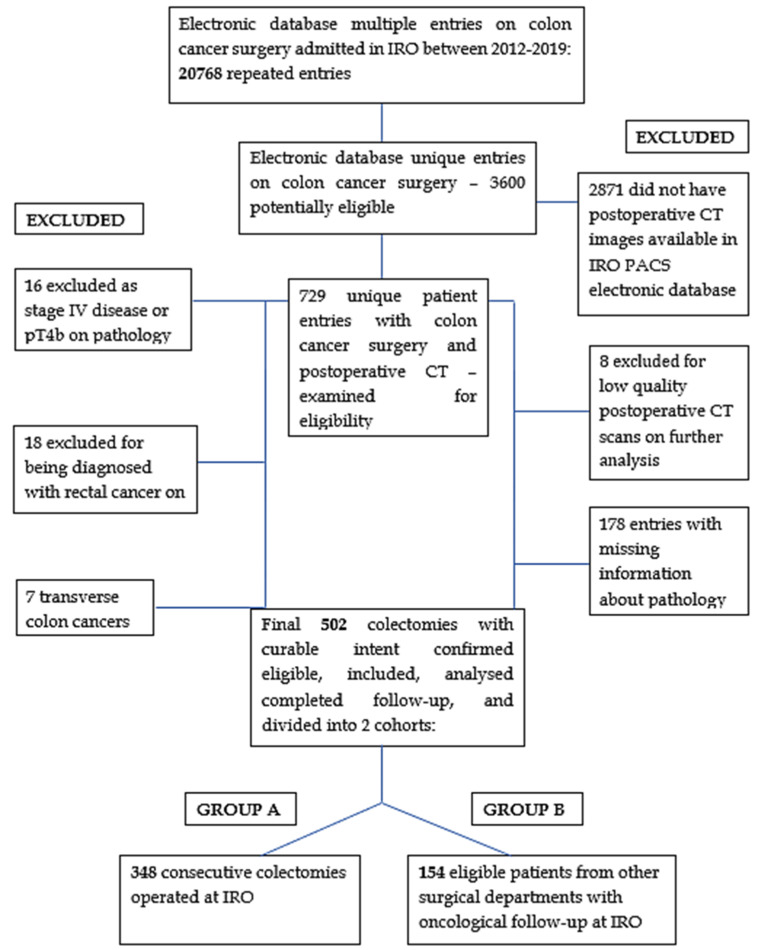
Flow diagram of eligibility.

All 3600 unique entries were individually analyzed for inclusion and exclusion criteria. Of these, 502 passed all the eligibility criteria (Figure 1) and were recorded with the following information to input in the analysis score: age at surgery, surgical department, colectomy date and type, eligible post-operative CT scan, time interval from surgery to post-operative CT, arterial stump angiometry parameters consistent with previous work published from our workgroup (actual stump length; theoretical best D_2_ position of predicted ligation level (D_2_PLL); theoretical best D_3_ position of predicted ligation level (D_3_PLL); D_2_ improvement potential (D_2_IP); D3 improvement potential (D_3_IP)) [9], pathological specimen parameters, established as independent prognostic factors for LRR [24,40,41] (pTNM stage, histological type of cancer; degree of differentiation; number of LNs, lymph-node index (LNI), expressed as the ratio between positive and total LNs; perineural [42], lymphatic and vascular invasion; tumor deposits (pN1c)), total follow-up interval, presence and type of LRR, and LRR-free survival (LRFS). The primary long-term oncological outcome was the LRFS.

Although LRR still lacks a clear definition [23], for the purpose of this study, LRR was considered to be tumoral growth detected by CT, magnetic resonance imaging, positron emission tomography-CT, colonoscopy, or intra-operative examination, in the following anatomical locations: peri-anastomotic site, mesocolic dissection margins, mesocolon or mesenteric fat, mesocolic or mesenteric LNs or central vascular LNs alongside the stumps of the ICA, right colic artery, sigmoid trunk, LCA, IMA, or in contiguity up to the lateral wall of the aorta. However, peritoneal carcinomatosis was not included in LRR; considering its multifactorial mechanisms, including hematogenous dissemination and frequent association with other metastatic sites, it does not reflect an exclusive failure of the surgical procedure. As such, patients with pT4a stage were included, with the specification that, in case of LRR, the score did not include cases of peritoneal carcinomatosis, as these were primarily excluded from the definition. Nevertheless, albeit locally advanced, pT4a cases are still surgically curable in stage III. Given the fact that the endpoint of this study was investigating LRR as defined above, only the locoregional dissemination mechanisms that would have been inherently caused by suboptimal surgical gestures were strictly regarded.

### 2.4. Image Acquisition Protocol

The technical aspects of the imaging protocol for the patients scanned at the IRO were consistent with previous work published from our workgroup [9]. The radiology department examined 400 patients included in our study on a BrightSpeed 16SL CT scanner with 16 detectors (GE Healthcare, Waukesha, WI, USA). The imaging station’s protocol included a 16 mm × 1.25 mm increment, pitch value of 1.75, table speed of 35.0 mm per rotation, tube output of 120 kV and 260 mA, with normal variations according to patient particularities. Multiphasic sectional imaging was captured after injecting Iomeron 400^®^ 8165 mg/mL (Bracco Imaging, Milano, Italy) with an automated infusion pump at a rate of 2.50 mL/s. Arterial and venous phases were obtained with an acquisition delay of 15 s or 50 s using the automated bolus tracker. Data sets were reconstructed with a 1–3 mm section thickness, and the reconstruction interval varied between 1 mm and 3 mm [9]. The remaining number of patients were scanned in other radiology departments with similar CT acquisition protocols, as is routine for CC follow-up.

### 2.5. CT Angiometry Protocol

A dedicated software package for radiological examinations was used (Radiant DICOM Viewer v.2020.2.3, Poznan, Poland) with 3D multiplanar reconstructions and maximum intensity projection tools for vascular identification. Arterial length measurements were standardized using the dedicated software instrument, conducted by the primary investigator (CL). Relevant CT sections and reconstructions were classified and saved in accordance with each designated clinic as proof of arterial stump measurement. The CT scans had been originally used for routine oncologic follow-up and were not ordered for the purpose of this study.

**Figure 2:**
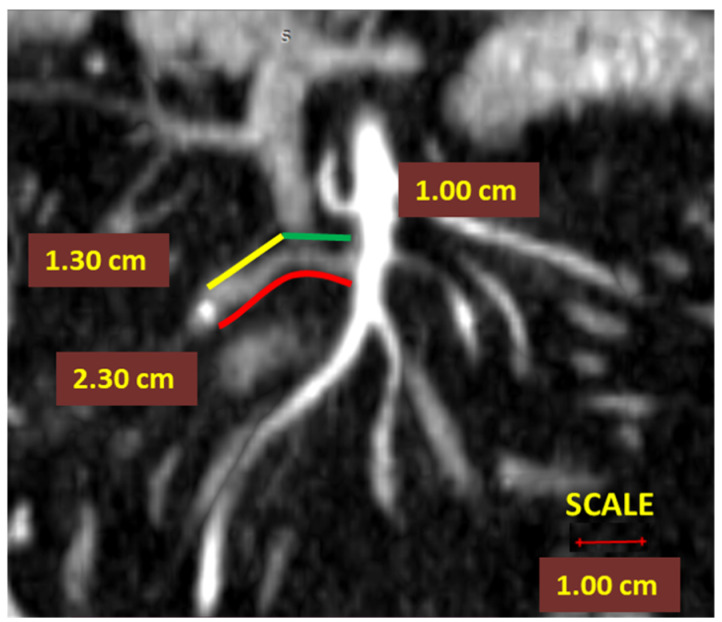
Computed tomography angiometry of the ileocolic artery stump after high ligation. The actual ileocolic artery (ICA) arterial stump length (ASL) is indicated by the red line. The theoretical best predicted ligation of ICA for D_2_ position of predicted ligation level (ICA-D_2_PLL) is indicated by the green line. The ICA D_2_ improvement potential (ICA-D_2_IP)—yellow line.

**Figure 3:**
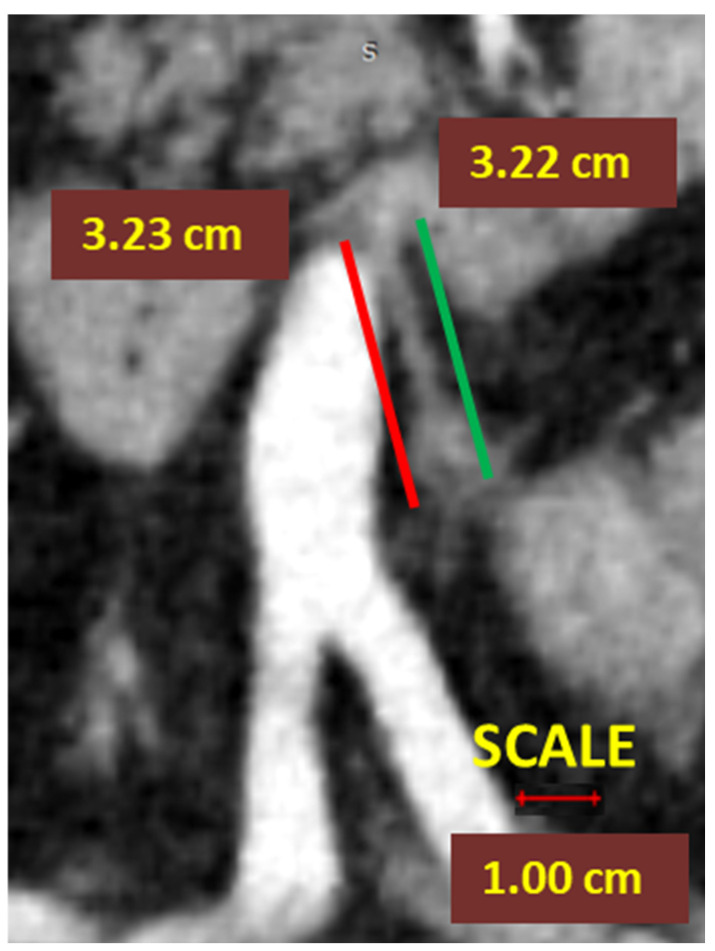
Computed tomography angiometry of the inferior mesenteric artery stump after D_2_ high ligation. The actual inferior mesenteric artery (IMA) arterial stump length (ASL) is indicated by the red line. The presumed IMA for D_2_ position of predicted ligation level (IMA-D_2_PPL) is indicated by the green line. The D_2_ improvement potential for ligation is reached.

**Figure 4:**
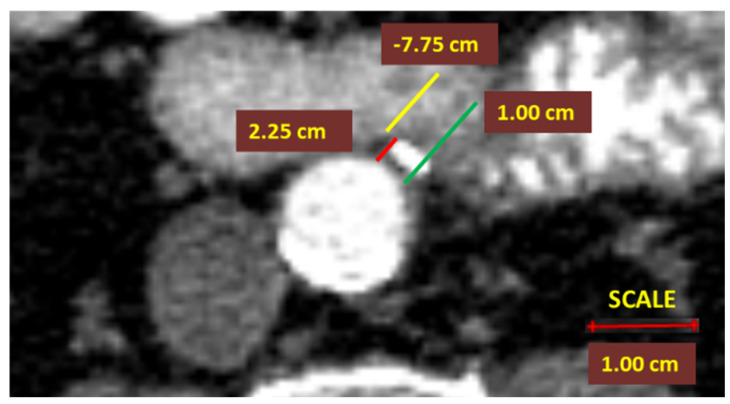
Computed tomography angiometry of the inferior mesenteric artery stump after D_3_ high ligation. The actual inferior mesenteric artery (IMA) arterial stump length (ASL) (red line) is 2.25 mm, ligated above the 10 mm D_3_ position of predicted ligation level (D_3_PPL) (green line), resulting in a D_3_ improvement potential (D_3_IP) of −7.75 mm (yellow line).

In the previous study of this workgroup, all angiometry measurements were validated through blinded evaluations by three independent radiologists. The level of inter-observer agreement, assessed for the three observers with the Kruskal–Wallis test, revealed no significant difference between data sets [9]. As defined in our previous study [9], the following quantitative angiometry parameters were extracted from each CT scan of the patients included in the study (actual ASL, D_2_PLL, D_3_PLL, D_2_IP, D_3_IP) as follows:

- In right colectomies, the ASL was defined as the length from the starting point of the ICA from the superior mesenteric artery to the ligation point. The presumed arterial length was defined as the length from the origin of ICA to the lateral side of the SMV, as benchmark length for D_2_ ligation, D_2_PLL (Figure 2);

- In left-sided colectomies, the ligation point was analyzed in relation to the IMA and the LCA. The ASL was measured from the origin of the IMA to the ligation point, while the presumed stump length (D_2_PLL) was measured from the IMA origin to below the LCA emergence, explicitly as the norm D_2_ lymphadenectomy level with conservation of the LCA. The D_2_IP was defined as the subtraction between actual stump lengths and the D_2_PLL (Figure 3).

- In contrast, for the D_3_ ligation level, a 10 mm benchmark was recommended as the presumed stump length in left colectomies [2]. The D_3_IP was calculated only for the IMA and represented the difference between actual stump lengths and the D_3_PLL approved by unanimity [2] (Figure 4);

- Middle and right colic artery stumps were not analyzed in this study due to the small sample size. The right colic artery was inconsistent and presented multiple anatomical variants. The middle colic artery was not routinely ligated for a standard right hemicolectomy.

### 2.6. Statistical Analysis

Continuous variables were presented as mean ± standard deviation (SD), median and the values of quartiles Q25 and Q75. Tests were applied to verify the type of distribution (Kolmogorov–Smirnov test) and for the homogeneity of the variances of the analyzed numerical value series (Levene’s test). Depending on the characteristics of the numerical values series, parametric (Student’s t test) or non-parametric (Mann–Whitney U Test, Tukey’s HSD for unequal N) comparison tests were applied. The Pearson chi-square test was applied to test the association of categorical variables. For testing the relation between two numerical variables, the Pearson correlation test was applied, and for qualitative variables of the ordinal type, the Spearman correlation test was applied. LRR predictability based on the analyzed values was evaluated based on the receiver operating characteristic (commonly known as ROC) curve and the area under the ROC curve (AUC) value. A risk score was generated for predicting LRR based on the logistic regression models that included certain explanatory variables. Based on this score, the probability of a patient presenting with LRR could be estimated. To validate the risk score, the internal validation bootstrap method (based on 2000 bootstrap samples) was applied. The reference threshold for the significance level *p*-value was considered to be 0.05. A value of *p* lower than 0.05 indicated with 95% confidence that there was statistical significance.

## 3. Results

A total of 502 patients out of 3600 were statistically analyzed. Baseline demographic and pathologic characteristics of patients are listed in Table 1 and Table 2.

**Figure 5:**
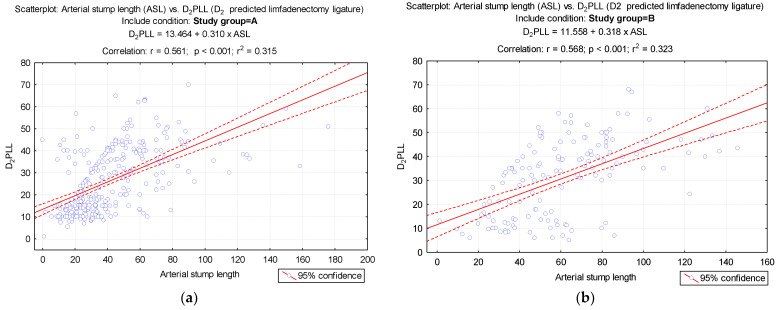
Evaluation of the correlation between arterial stump length and D_2_ lymphadenectomy. (**a**) Group A has a compact relationship of arterial stump length (ASL) and D_2_ position of predicted ligation level (D_2_PPL) showing a strong positive correlation; (**b**) Group B relationship of ASL and D_2_PPL showing positive correlation but with wide dispersion of values.

Figure 5 shows the strong correlation between the ASL and D_2_PLL values from each group. The compact and narrow distribution of ASLs from Group A is to be noted in contrast with the wide dispersion of ASL from Group B (Figure 5). 

The median follow-up interval was 23 months, and LRR arose after 12 months. ASL stratified by colectomy type was significantly smaller in Group A. The mean ASL (± SD) showed significantly higher values, 50.8 mm ± 28.5 mm in the LRR group vs. 45.5 mm ± 28.1 mm in the group without LRR (*p* < 0.001; Table 3). The overall rate of LRR was statistically significant between groups, with 11.5% in Group A and 24.0% in Group B (*p* < 0.001).

### 3.1. LN Number below 12 Correlates with LRR

**Figure 6:**
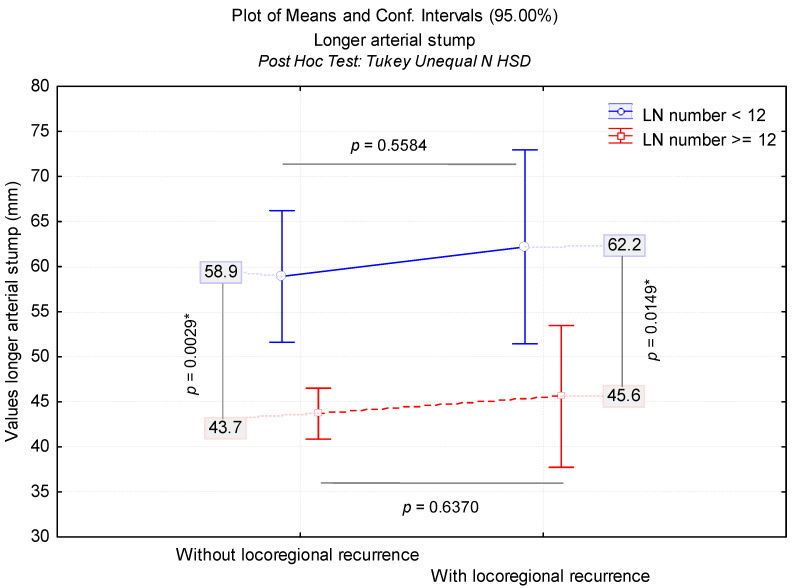
Comparison of arterial stump length values according to locoregional recurrence and lymph node number (below and above 12). LN: Lymph node; LRR: Locoregional recurrence. * Marked effects are significant at *p* < 0.05.

In total, 77 (15.3%) out of 502 colectomies resulted in an LN count below 12. This accounted for 53 cases (34.4%) from Group B and 24 cases (6.9%) from Group A. A comparison between the two groups revealed that Group B consisted of a statistically higher frequency of cases with less than 12 LNs (*p* < 0.001). There was a strong, statistically significant correlation between the LN number < 12 (*r* = −0.486, *p* < 0.001) and the presence of LRR, as 24 out of the 77 cases (31.2%) had LRRs. These patients (subgroup with LNs < 12 with LRR) illustrated a significantly longer ASL, with mean 62.2 mm, and 22 out of 24 cases having left-sided CCs (Figure 6, Table 4). Furthermore, 27 surgical specimens yielded less than 6 LNs, with 24 being from Group B colectomies.

**Figure 7:**
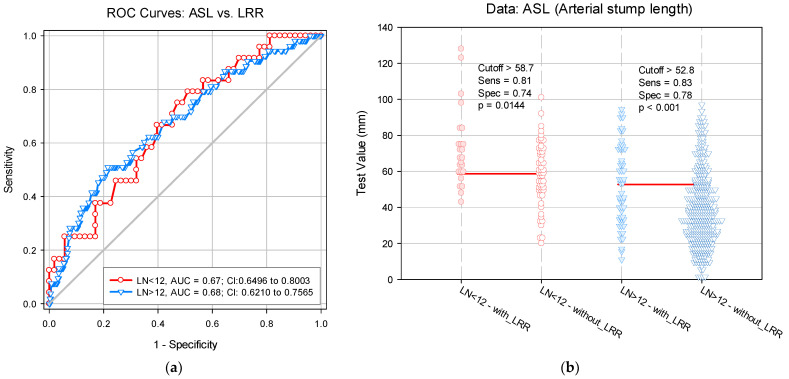
(**a**) Receiver operating characteristic curve. (**b**) Paired histogram for estimating the arterial stump length cut-off value for locoregional recurrence predictability. LN: Lymph node; LRR: Locoregional recurrence; Sens: Sensitivity; Spec: Specificity.

The risk assessment of LRR was analyzed according to ASL and LN number. In this context, we estimated the ASL cut-off value for which the LRR risk increases significantly. Given the significant association between LRR and the LN number, we evaluated the ASL cut-off value adjusted for LN count. The results indicated an ASL cut-off value of 58.7 mm (*p* = 0.014) for LN values < 12 and an ASL cut-off value of 52.8 mm (*p* < 0.001) for LN values > 12. This reference threshold is a good risk predictor, as shown by the area under curve: AUC for LN < 12 = 0.67; AUC for LN > 12 = 0.68 (*p* < 0.001). The predictive value of the proposed ASL reference does not show a significant difference between the specificity and sensitivity of the evaluation method for LRR prediction (*p* = 0.9650), depending on the adjustment variable (Figure 7).

**Figure 8:**
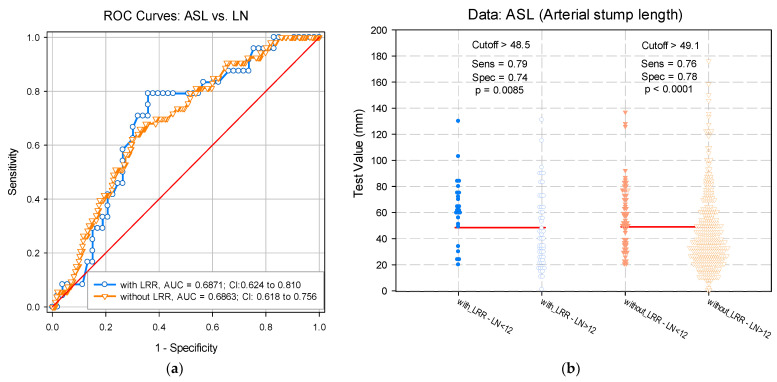
(**a**) Receiver operating characteristic curve. (**b**) Paired histogram for estimating the arterial stump length cut-off value based on predictability of lymph node number. LN: Lymph node; LRR: Locoregional recurrence; Sens: Sensitivity; Spec: Specificity.

Alternatively, the LRR risk assessment was analyzed according to the LN number (greater or lower than 12) with the calculated ASL cut-off value being generated by applying the adjustment after LRR. Thus, the cut-off value of ASL was calculated to assess the risk of LNs < 12 in the presence of LRR (ASL_cut-off_ = 48.5 mm; *p* = 0.0085) and in the absence of LRR (ASL_cut-off_ = 49.1 mm; *p* < 0.001). Similarly, no significant differences were identified between the ASL cut-off predictive value calculated using the LRR adjustment variable (*p* = 0.991) (Figure 8).

### 3.2. Risk Score Calculation

The predictive value of the parameters for predicting LRR after surgical treatment of CC was evaluated based on the univariate and multivariate logistic regression model (Table 5). The first stage was based on univariate analysis (logistic regression), and only statistically significant predictors were included in the risk model.

To create a risk score, the rule based on the ratio between the numbers of events per variable (prediction factor) was considered. This requires a minimum of 10–15 events for each predictor entered in the model.

To reduce the number of predictors before creating the risk score, the stepwise selection model (Backward Stepwise—Wald) was applied in the second stage, in which non-significant predictors had been gradually removed from a model that initially contained all candidate predictors. Subsequently, for establishing the risk score, the internal bootstrap validation was applied. This way, the variables that did not have a significant contribution to the risk score were excluded from the model.

All steps corresponding to the model validation were followed (four steps). Before using the risk model, the predictive capacity of the model was evaluated—more precisely, the validation of the model. This involved an assessment of calibration (agreement between observed results and predictions) and discrimination (the model’s ability to discriminate between low- and high-risk patients). Subsequently, the model was internally validated using bootstrapping. Based on the constant model (without predictors), 84.7% of cases could be correctly anticipated from the LRR point of view. At this stage, the contribution of each independent variable to the improvement of the model was evaluated.

Only the last step (step 4) was presented in establishing the predictor variables that would be subsequently included in the risk score. Variables not statistically significant were excluded from the model (Table 6).

In order to be considered with good predictive power, the significance of the proposed model was evaluated. The results indicated that the model was able to correctly evaluate a significant number (*p* < 0.001) of cases, with the difference between the complete model and the model without predictors being small (−2LL = 18.15). Nagelkerke R Square estimated the share of cases for which the evolution could be explained based on the chosen model as 86.2%.

Based on the results of the Hosmer–Lemeshow test, the model was fit and did not show a significant difference between the real case evolution and the one estimated by it. The model calibration results for the analyzed data (Hosmer–Lemeshow test) indicated that the estimated frequency of LRR was not significantly different from the estimated model (χ^2^ = 9.22, *p* = 0.101), confirming that the generated model was appropriate.

Based on the obtained results, the proportion of correctly classified cases following the introduction of predictors was 92.6%. Practically, this result demonstrated a significant improvement of the model with predictors (84.7% vs. 87.5%).

The internal validation highlighted that the model was correct and coefficients’ values remained constant after bootstrapping had been applied. Based on the model defined in this way, the probability of LRR for all patients with CC was estimated (Table 7 and Table 8).

Patient’s risk of LRR is calculated as follows:

$$Patient’s\;risk\;LRR=\frac{\;exp^{\left(\mathrm{intercept}\;+\left(\beta_{GRP}\times \;\mathrm{GRP}\right)+\left(\beta_{ASL}\times \;\mathrm{ASL}\right)+\left(\beta_{index}\times \;\mathrm{LNI}\right)\right)}}{1+exp^{(\mathrm{intercept}\;+\left(\beta_{GRP}\times \;\mathrm{GRP}\right)+\left(\beta_{ASL}\times \;\mathrm{ASL}\right)+\left(\beta_{index}\times \;\mathrm{LNI}\right)}}$$

The variables corresponding to the previous formula are:

Intercept = 0.215—the corresponding value in Table 8;

β_GRP_ = 0.596 (Table 8);

GRP = 1 if the patient is in Study group B and GRP = 0 if the patient is in Study group A;

β_ASL_ = 0.101 (Table 8); ASL—the arterial stump length of the patient;

β_LNI_ = 2176 (Table 8); LNI—lymph node index of the patient.

Patient’s risk of LRR was defined as the probability of a case to be in a certain risk category. Thus, based on the risk score calculated according to the predictor variables, for each patient the probability that they would have LRR was calculated.

Based on the risk scores created, the patients were stratified into three risk categories. In Table 9, each risk category was verified to be associated with the presence or absence of LRR. If the prediction score had been inappropriate for testing our hypothesis, the ranking categories would not have correlated with the LRR. 

Depending on the calculated risk for each patient, patients were divided into three risk categories: low risk, for a calculated risk in the range 0.25–0.5; moderate risk, for a calculated risk in the range 0.5–0.75; and, high risk, for calculated risk values > 0.75.

The results showed a very strong correlation between the risk categories created based on recurrence predictive factors (study group, ASL and LNI) and the patient LRR itself. This was clearly demonstrated by the fact that, in patients without LRR, the generated risk category based on the prediction score placed 87.76% of patients at low risk. On the other hand, the prediction score placed 76.6% of patients with LRR in the high risk class. The strong, significant correlation (*r* = 0.741, *p* < 0.001) proved the highly predictive value of the created risk score (Table 9).

**Figure 9:**
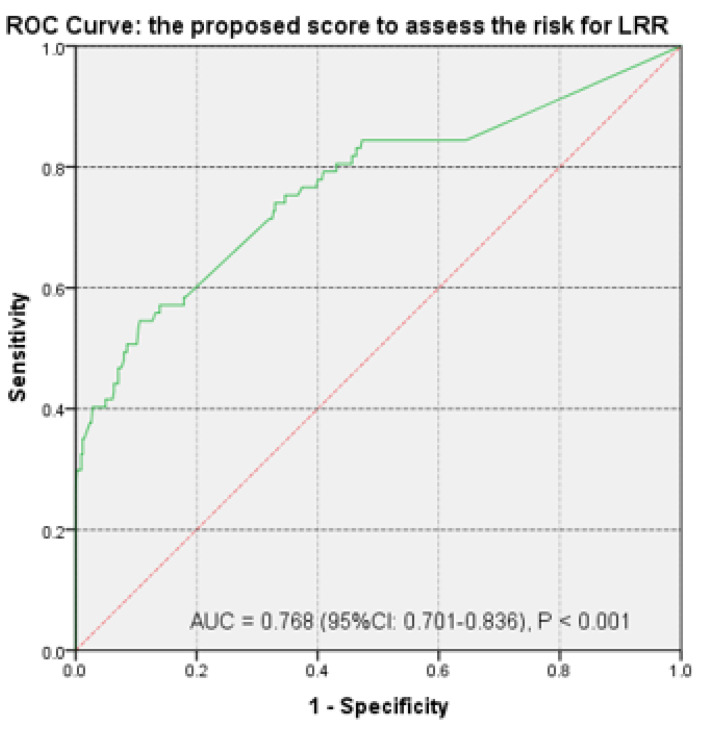
Receiver operating characteristic curves for evaluation of the predictive power of locoregional recurrence based on the proposed score. AUC: Area under the curve; LRR: Locoregional recurrence; ROC: Receiver operating characteristic.

At the same time, the ROC curve was analyzed for the new predicted probability scores to evaluate their predictive value and accuracy. The AUC value confirmed the increased predictive power. Thus, the created score provided very good discrimination for differentiating patients at risk for LRR (Figure 9).

The main long-term oncological result was analyzed: LRFS, which was compared between the two study groups (Group A, Group B). 

**Figure 10:**
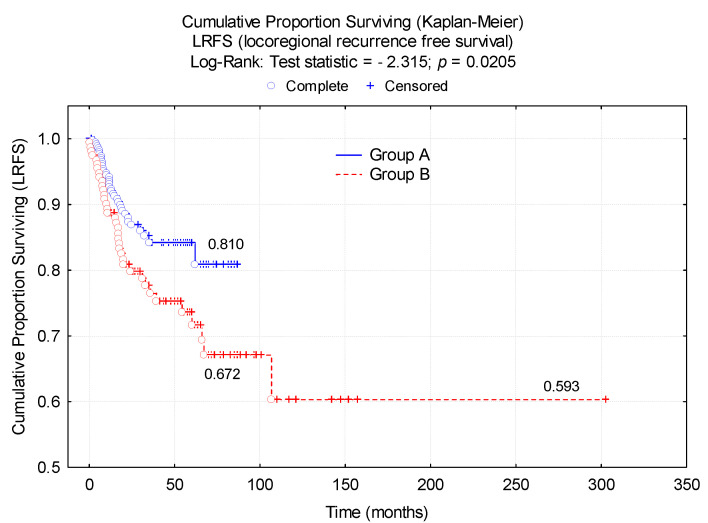
Locoregional recurrence-free survival by study group. Kaplan–Meier chart.

The results indicated a significantly higher LRFS probability (*p* = 0.02) in Group A (81%) compared to Group B (67.2%) at 67 months after surgery (Figure 10).

## 4. Discussion

Our analysis showed an overall LRR rate of 15.6% at 37 months and, when stratified between the two cohorts, an 11.5% LRR rate for Group A and 24% LRR rate for Group B. The LRR after curative treatment for CC is 11.5% as extracted from the National Swedish registry [23], similar to the LRR rate of Group A in our study. According to our Kaplan–Meier analysis, there was a significant difference (13.8%) in LRFS at 5.58 years, when CME was performed (81%), in contrast to non-CME surgery (67.2%). The LRR difference alone between groups A and B would be sufficient to argue that standardized CME with constant rigorous vascular ligation ought to be the norm and best practice, to reduce the dramatically high rates of LRR derived from suboptimal surgical gestures [13]. When Bertelsen et al. [2] compared oncological outcomes after CME colectomies performed at one hospital with non-CME colectomies performed at three other hospitals, they also reported a 2.9% decrease in local recurrence at 5 years, demonstrating that the mesocolic resection technique is an independent factor for positive prognosis in all resectable patients. Additionally, West [29] suggested that standard segmental colectomy presents a variable quality and resection of the mesocolon in the extrafascial plane improved the 5-year prognostic up to 27% in node-positive patients [43].

The comparative representation of each measured ASL, represented in the scatterplot from Figure 5, aids illustration of intrinsic surgical technique variations between CME and conventional colectomies. The compact distribution of ASL from Group A with focal density towards the smaller values is confirmation of a meticulous and disciplined CME surgery with constant CVL, whereas Group B ligation heights follow a wider dispersion, reflecting suboptimal excision.

The cut-off value of 12 LNs is still considered the minimum quality benchmark for adequate nodal clearance and determines adjuvant treatment [24]. Our study discovered 77 out of 502 colectomies with LN count under 12. Of these, 53 were from Group B and 24 from Group A. The LRR rate in Group B patients was 24 out of 77 (31%), and these illustrated a significantly longer arterial stump (mean of 62 mm; 22 being left-sided CCs). These results support the argument that an ASL over 62 mm correlates with a poor LN yield and a very high risk of LRR, up to 31%. This suggests that a second surgical procedure for completing the mesocolic excision height around the arterial stump would be oncologically beneficial for one in three patients and would provide correct nodal staging in the other two, thus allowing for accurate selection of patients for adjuvant chemotherapy [44].

Our results suggest that, after performing the oncologic follow-up CT, a thorough multidisciplinary evaluation score should be calculated to stratify patients according to the LRR risk. All efforts should be directed towards prevention of recurrence, either by a second surgical procedure or adjuvant chemotherapy, as Jung showed that survival sharply decreases after recurrence even if surgical resection is curatively performed [45]. The literature shows that failing to resect an LRR in a timely manner is in most cases a failure to resect at all, as only 12% of LRRs presented by Sjövall [23] could have been resected curatively. The authors showed that out of 192 patients with LRR, surgery was performed in 57% but only 23 (12.0%) had a potentially curative resection. Another 13% received palliative chemo-/radiotherapy, and just palliative care was offered to approximatively 30%. Surgical interventions were performed with significantly higher mortality (14.6%) in palliative procedures compared with curative surgeries (4.8%) [23,46]. 

Fuzun [47] showed that no significant differences in survival were obtained after R0, compared to R1 salvage surgery for LRR after cancer colectomies. However, significant benefits in survival occurred after R1, as opposed to that after R2 surgery. The authors recommended that at least R1 clearance should be obtained. Information on LRR management clearly exemplifies that a prophylactic surgery would be most indicated before the LRR can become unresectable. Even if R0 is performed, the best survival benefit still does not surpass that of R1 resection.

According to our patient demographics, stage III CC was statistically prevalent in our database. This was due mainly to the lack of a national screening system for colorectal cancer, although some local screening protocols have been implemented by our institute. As shown in the literature, the countries that implemented a systematic, nationally funded colorectal screening program successfully decreased the incidence of stages III and IV and increased the rate of stage I and II stages from 10.1% before screening to 45.3% after screening. Considering that stage I and II have a < 10% risk of nodal disease, a systematic D_3_ lymphadenectomy would be a too-severe surgical intervention that would subject most patients in the general population to an unbalanced risk-benefit ratio [41,48]. On the contrary, the stage distribution in countries without national screening, such as ours, is in favor of stages III and IV, with a spike in stage III [49]. Hence, it may be possible that D_3_ lymphadenectomy could bring some benefit in selected cases, at the expense of higher perioperative risks. However, standardization of D_2_ lymphadenectomy for all colectomies, tailored to country stage distribution frequency, would offer the greatest benefits in terms of a larger count of negative nodules and a better ratio of positive/total LNs [50,51,52,53].

Regarding lower LN yields after sigmoidectomy and segmentectomy for left-sided CCs, the mean LN count in Group B was 15 LNs, compared to 28 LNs in Group A. The main argument for this would be the constant, sub-segmentary, distal ligation of the inferior mesenteric branches, with mean ASL of 71 mm in Group B vs. 51 mm in Group A. These findings are similar to those of Sjövall [23], where significantly fewer LNs were examined for sigmoid cancer compared to all other locations.

**Figure 11:**
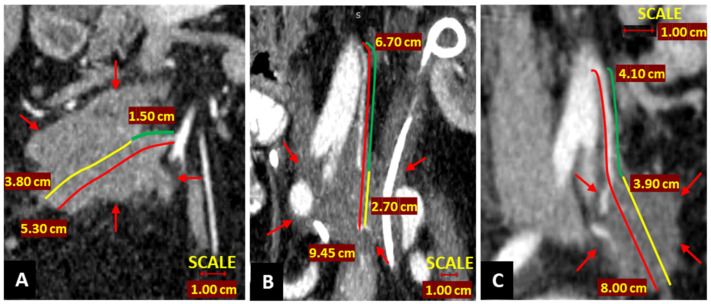
Postoperative computed tomography in coronal reconstruction, illustrating large regional confluent adenopathies (marked with red arrows) enclosing the residual colic arteries after low ligation. (**A**) Post-right hemicolectomy ileocolic artery (ICA) arterial stump length (ASL) (red line) measuring 5.30 cm, presumed D_2_ position of predicted ligation level (D_2_PPL) of 1.5 cm (green line) and D_2_ improvement potential (D_2_IP) of 3.8 cm (yellow line). (**B**) Tumoral enclosing of the left ureter and inferior mesenteric artery (IMA), which is ligated below the D_2_ benchmark after sigmoidectomy, with IMA ASL measuring 94.5 mm (red line), the presumed D_2_PPL, 6.70 cm (green line) and the D_2_IP, 2.70 cm (yellow line). (**C**) Post-sigmoidectomy IMA ASL measuring 8.00 cm (red line), the presumed D_2_PPL, 4.10 cm (green line) and the D_2_IP, 3.90 cm (yellow line).

A sub-analysis was performed on the difference in LNs and ASL after group A D_3_-left-hemicolectomy and D_2_-left-hemicolectomy, revealing an average addition of 10 LNs along a mean arterial length of 26 mm when performing a high ligation for left hemicolectomy as compared to a low ligation. Grinnell [54] also documented that this arterial segment carries up to 10 LNs. Rosi [55] demonstrated that up to 22% of sigmoid cancers have positive LNs at 10 mm to 30 mm from the IMA origin, with impact on survival. Furthermore, Andrew [41] appreciated that positive apical nodes contribute up to 25% of cases. Regarding this, Rao et al. [56] argue that positive nodes located above the origin of the LCA represent an independent risk factor for metastatic disease, contributing to the importance of the LN clearance along the IMA stump. Various other studies have reported high regional recurrences around the ileocolic, superior mesenteric and inferior mesenteric arteries, as higher regional recurrences were caused by residual LNs in these key sites, whereas clearance of these LNs prolonged OS [23,57]. Figure 11 depicts the consequences of leaving behind long post-colectomy arterial stumps (Group B): large adenopathic recurrences encasing the residual stumps (Figure 11). In addition, according to Lips [58], LRR or systemic metastasis will occur in approximately one-third of node-negative CC patients with curative excision. They attribute this to occult micro-metastases or isolated tumor cells and recommend broadening the indication for post-operative chemotherapy to include these cases. To this end, CME with standardized CVL would improve accurate nodal staging by including more conventionally negative LNs that could harbor micro-metastases and isolated tumor cells, in order to categorize patients for adjuvant treatment [58,59].

### Limits

Regarding the time from surgery to local relapse, LRR occurs rather early in the evolution of disease, at a median of 12 months in our study and up to 3 years in the literature [23]. Further, considering that the incidence of LRR correlates with systemic disease at 5 years, our follow-up interval of median 23 months was considered to be adequate for observing the rate of LRR [12]. LRR is most frequently noted in the initial years after radical colectomy [60]. Additionally, a 3-year DFS is acknowledged as a pertinent representative endpoint for a 5-year OS in CC studies [61,62,63]. 

Considering that the time from surgery to postoperative CT was statistically different between the two groups (mean of 12.7 months in Group A and 26.2 months in Group B; *p* < 0.001), some may argue that this may impact stump length. However, there are studies that have clearly shown post-colectomy arterial stumps over 2 years after surgery [38]; hence, this variable would have brought insignificant errors upon our final results. Nevertheless, even if the ASLs from Group B had been affected by retraction over time, this would have only caused a smaller difference in ASL comparison between groups, with the difference retaining statistical significance.

Emergency surgery was not included as a variable in our analysis. Emergency surgery has been proposed as an independent negative prognostic factor by some studies, while most studies dispute that the urgent surgery itself is not a risk factor but, more precisely, the clinical circumstances would cause worsening of oncologic objectives [19,64,65]. Antony [66], using propensity score risk adjustments, as well as Guidolin [67], showed no attributable oncologic risk factor for emergency surgery of CC. Nevertheless, a complete tumor and mesocolic excision at initial surgery remains primordial for the overall outcome [23,64]. Although our study design was retrospective, the data had been registered prospectively. Surgical teams from Group B were inhomogeneous, but they reflect real-life practices in the northeastern region of Romania, which our IRO serves.

Some population-based investigations of recent date indicate that women with colon cancer exhibit better overall survival compared with men, due to complex factors such as estrogen exposure or tumor location, without significant differences in disease stage or therapeutical management [68]. Although an overall 55% male predominance was obtained in our study, this confounding variable was not included as a risk factor in the final prognostic score. 

Several biomarkers have been postulated with their respective prognostic significance, but with limited changes in practice. Inflammatory cytokines such as IL−6, CRP and sCD40L have been shown to reflect the progression of colorectal cancer and predict tumor relapse, but most of them are postulated as indirect surrogate prognosis factors due to their correlation with already established WHO pathological grading criteria [69]. Regarding microsatellite instability (MSI), a screening study of deficient mismatch repair profiles on colorectal cancer distribution in Romania by Lungulescu showed that MSI status does not carry a significant prognostic role [70]. Apart from clinical and pathological factors, new insights regarding the role of genetic [71], transcriptomic [72] and mass spectrometry imaging proteomics analysis [73] as prognostic instruments are still under investigation concerning the influence upon clinical decision in loco-regional recurrence prediction. Additionally, immunohistochemical expression of the REG4 proteins, frequently detected in right colon cancer, was significantly correlated with low recurrence-free survival [74]. Although we recognize their potential prognostic role, inflammatory biomarkers, MSI status, genetic profiling, transcriptomics, proteomics, or REG4 status were not accounted for in our prognostic score due to their diverse and inhomogeneous usage in everyday clinical practice.

Regarding specimens with pT4a on pathology after curative resection, they were included only if the LRR mechanism was assumed to be surgically derived, such as anastomotic relapse or LN recurrence. Some may argue that, in pT4a, peritoneal contamination may have already occurred before the primary surgery; however, patients that relapsed with isolated peritoneal carcinomatosis were excluded. In other words, whenever the mechanism of relapse favored hematogenous or transperitoneal spread of cancer cells, such as in the case of peritoneal carcinomatosis, corresponding pT4a patients were excluded, supporting our aim to identify surgical procedure failures and potential solutions.

## 5. Conclusions

We developed and validated an LRR prediction score. The LN index, the type of surgery (CME or conventional) and the ASL were the statistically significant variables that constructed the score. CME provides surgical specimens superior in quality, with lower LRR rates and longer LRR-free survival in contrast to conventional unstandardized colectomies. The ASL has been shown to significantly differ between CME and conventional colectomies. The cut-off value of the arterial stump could be informative for the multidisciplinary team, when evaluating the need for re-surgical completion of the mesocolic excision, if integrated in the validated LRR score. If a patient with a long residual vascular stump presents other known risk factors, a second surgery could provide prophylaxis of the recurrence in due time, before it occurs.

## Figures and Tables

**Table 1:** Baseline demographic and pathologic characteristics of patients.

Characteristics	Group A	Group B	*p*-Value
*n* = 502	*n* = 348	*n* = 154	
Age at surgery, in years Mean ± SD	64.2 ± 10.2	59.5 ± 10.3	<0.001
Median (Q25; Q75)	66 (58.5; 71)	60 (54; 67)	
Sex	190/158	89/65	0.506
Male/female, *n* (%)	(54.6%/45.4%)	(57.7%/42.2%)	
Type of colectomy, *n* (%)			
Right hemicolectomy	139 (39.9%)	44 (28.6%)	0.092
Extended right hemicolectomy	10 (2.9%)	5 (3.2%)	
Left sided colectomies	199 (57.2%)	105 (68.2%)	
Left hemicolectomy	40 (11.5%)	14 (9.1%)	
Extended left hemicolectomy	2 (0.6%)	2 (1.3%)	
Sigmoidectomy	135 (38.8%)	80 (51.9%)	
Segmentectomy for left colon flexure	22 (6.3%)	9 (5.8%)	
pT stage, *n* (%)			
pTx	1 (0.3%)	0 (0%)	0.018
pTis	1 0.3%)	1 (0.6%)	
pT1	10 (2.9%)	0 (0%)	
pT2	27 (7.8%)	11 (7.1%)	
pT3	236 (67.8%)	94 (61%)	
pT4a	73 (21%)	48 (31.2%)	
pN stage, *n* (%)			
pNx	0 (0%)	4	0.008
pN0	166 (47.7%)	66	
pN1	119 (34.2%)	48 (31.2%)	
pN1apN1b/pN1c	63 (18.1%)/49 (14.1%)/7 (2%)	15 (9.7%)/31 (20.1%)/2(1.3%)	
pN2	63 (18.1%)	36 (23.4%)	
pN2a/pN2b	40 (11.5%)/23 (6.6%)	14 (9.1%)/22 (14.3%)	
M (metastasized) stage M0/M1, *n*	348/0	154/0	
LRR	40 (11.5%)	37 (24.03%)	
Time (Op-LRR), months			0.198
Mean ± SD	13.1 ± 8.2	23.1 ± 25	
Median (Q25; Q75)	12 (7–16.8)	16 (8–29)	
Time (Op-CT), months Mean ± SD Median (Q25; Q75)	12.7 ± 7.92 8.25 (6; 15)	26.2 ± 17.08 12 (6; 37.5)	<0.001
Histology			
Adenocarcinoma only	265 (76.2%)	119 (77.3%)	0.816
Adenocarcinoma with mucinous cell carcinoma component	77 (22.1%)	31 (20.1%)	
Adenocarcinoma with signet cell carcinoma component	4 (1.2%)	2 (1.3%)	
Adenocarcinoma with both mucinous and signet cell carcinoma components	2 (0.6%)	2 (1.3%)	
ASL in mm			<0.001
Mean ± SD	40.04 ± 25.34	60.72 ± 28.87	
Median (Q25; Q75)	33.25 (23.25; 51.6)	58 (37.6; 80)	
D_2_PLL in mm			<0.001
Mean ± SD	26.2 ± 14.4	31.2 ± 16.0	
Median (Q25; Q75)	23 (13.5; 38.4)	34.9 (14; 42.8)	
LN(s) < 12, *n* (%)	24 (6.9%)	53 (34.4%)	<0.001

ASL: Arterial stump length; CT: Computed tomography; D_2_PLL: D_2_ position of predicted ligation level; LRR: Locoregional recurrence; LN(s): Lymph node(s); Op-CT: Time interval from surgery to post-operative computed tomography; Op-LRR: Time interval from surgery to locoregional recurrence; Q25: Lower quartile; Q75: Upper quartile; Op: Surgery.

**Table 2:** Tumor-node-metastasis stage—Union for International Cancer Control 8th edition.

TNM Stage	Group A	Group B	*p*-Value
*n* (%)	*n* = 348	*n* = 154	
0	1 (0.29%)	1 (0.65%)	0.028
I	23 (6.61%)	7 (4.55%)	
II (II A/II B)	141 (122/19) 35.06%/5.46%	58 (47/11) 30.52%/7.14%	
III (III A/III B/III C)	184 (12/130/40) 52.87% (3.45%/37.36%/11.49%)	84 (1/54/29) 54.54% (0.65%/35.06%/18.83%)	
N/A (pTx or pNx)	1 (0.28%)	4 (2.60%)	

N/A: Not available; pNx: Regional lymph nodes cannot be evaluated pathologically; TNM: Tumor-node metastasis; Tx: The primary tumor cannot be evaluated pathologically.

**Table 3:** Descriptive statistics of arterial stump length between cohorts with and without locoregional recurrence.

LRR	Cases, *n*	ASL in mm							
		Mean (95%CI)	SD	SE	Min	Max	Q25	Median	Q75
No	425	45.5 (42.9–48.2)	28.0	1.3	0	176.0	26	39	59.4
Yes	77	50.8 (44.3–57.2)	28.5	3.2	1	131.0	26	48	70.0
All groups	502	46.3 (43.9–48.8)	28.1	1.2	0	176.0	26	40	61.0

Z_adjusted_ = 8.1799, *p* < 0.001. ASL: Arterial stump length; CI: Confidence interval; LRR: Locoregional recurrence; Max: Maximum; Min: Minimum; Q25: Lower quartile; Q75: Upper quartile; SD: Standard deviation; SE: Standard error.

**Table 4:** Descriptive statistics of arterial stump length in relation to locoregional recurrence and lymph node count (below and above 12).

LRR	LN(s)	Cases, *n*	ASL in mm						
			Mean (95%CI)	SD	SE	Min	Max	Median	Q25; Q75
No	<12	53	58.9 (51.6–66.2)	26.5	3.6	20	137	57	37; 76.6
	>12	372	43.7 (40.9–46.5)	27.7	1.4	0	176	37	25; 55
Yes	<12	24	62.2 (51.4–72.9)	25.5	5.2	20	130	61.6	50; 75
	>12	53	45.6 (37.7–53.5)	28.5	3.9	1	131	40	23.6; 63
All groups		502	46.4 (43.9–48.9)	28.1	1.3	0	176	40	26; 61

ASL: Arterial stump length; CI: Confidence interval; LN(s): Lymph node(s); LRR: Locoregional recurrence; Max: Maximum; Min: Minimum; Q25: Lower quartile; Q75: Upper quartile; SD: Standard deviation; SE: Standard error.

**Table 5:** Univariate and multivariate logistic regression for locoregional recurrence prediction.

Logistic Regression	Odds Ratio (95%CI)	SE	*p*-Value
**Univariate Analysis**			
Age in years	1.2 (1.1–1.5)	0.011	0.003
Study group B, ref. Group A	2.4 (1.5–3.9)	0.253	<0.001
Type of colectomy, ref. left sided colectomies Right hemicolectomy	1.1 (0.7–1.8)	0.252	0.680
Time (Op-CT) in months	0.99 (0.98–1.01)	0.06	0.852
Degree of differentiation	1.2 (0.7–1.9)	0.240	0.442
ASL	1.7 (1.1–5.1)	0.004	0.014
LNI	7.8 (3.9–9.5)	0.087	<0.001
Total LN(s)	0.97 (0.95–0.99)	0.009	0.002
LN(s) < 12	3.2 (1.8–5.6)	0.287	<0.001
Positive LN(s)	1.08 (1.03–1.14)	0.028	0.005
pT	1.7 (1.1–2.6)	0.211	0.011
pN	1.09 (0.81–1.48)	0.157	0.566
Histology, ref. adenocarcinoma only Adenocarcinoma with mucinous cell carcinoma component Adenocarcinoma with signet cell carcinoma component Adenocarcinoma with both mucinous and signet cell carcinoma components	1.5 (0.8–2.6) 3.1 (0.5–17.4) 2.1 (0.2–20.4)	0.285 0.879 1.164	0.149 0.195 0.529
Perineural invasion	1.8 (1.1–3.2)	0.280	0.025
Lymphatic invasion	0.7 (0.4–1.2)	0.263	0.180
Vascular invasion	0.9 (0.5–1.7)	0.270	0.960
Tumor deposits, pN1c	1.3 (0.5–3.4)	0.472	0.531
D_2_IP	1.01 (0.99–1.07)	0.005	0.187
D_3_IP	1.01 (0.99–1.01)	0.004	0.199
**Multivariate analysis, Method: Backward Stepwise** **Step 1**			
Study group B, ref. Group A	1.81 (1.02–3.2)	0.291	0.041
ASL	1.18 (1.01–3.6)	0.005	0.040
LNI	6.03 (1.6–12.6)	0.673	0.008
pT	1.3 (0.8–2)	0.220	0.225
Perineural invasion	1.5 (0.8–2.7)	0.303	0.189
**Step 4**			
Study group B, ref. Group A	1.82 (1.06–3.1)	0.274	0.029
ASL	1.1 (1.01–1.3)	0.005	0.043
LNI	8.8 (2.4−12.1)	0.644	0.001

ASL: Arterial stump length; CI: Confidence interval; D_2_IP: D_2_ improvement potential; D_3_IP: D_3_ improvement potential; LN(s): Lymph node(s); LNI: Lymph node index; Op-CT: Time interval from surgery to post-operative computed tomography; SE: Standard error.

**Table 6:** Strength assessment of variables with regard to score model contribution: Variables not in the model.

			Score	*p*-Value
Step 4	Variables	pT	2.184	0.139
		Perineural invasion	2.565	0.109
	Overall statistics		4.095	0.251

**Table 7:** Model coefficients and Wald test with regard to predictive factors for locoregional recurrence.

	Variables in the Equation	β	SE
Step 4	Study group B, ref. group A	0.596	0.274
	ASL	0.101	0.005
	LNI	2.176	0.664
	Intercept	0.215	0.180

ASL: Arterial stump length; LNI: Lymph node index; SE: Standard error.

**Table 8:** Bootstrap validation: Model coefficients regarding predictive factors for locoregional recurrence.

	Variables in the Equation Bootstrap ^§^	β	SE
Step 4	Study group B, ref. group A	0.596	0.241
	ASL	0.101	0.002
	LNI	2.176	0.017
	Intercept	0.215	0.205

^§^ Bootstrap results are based on 2000 bootstrap samples; for group A, complete mesocolic excision: GRP is 0; for group B (non-complete mesocolic excision): GRP is 1. ASL: Arterial stump length; LNI: Lymph node index; SE: Standard error.

**Table 9:** Patient stratification into created risk categories using the score of locoregional recurrence prediction.

	Low Risk	Moderate Risk	High Risk	Total
Without LRR	373 (87.76%)	43 (10.12%)	9 (2.12%)	425
With LRR	4 (5.19%)	14 (18.18%)	59 (76.62%)	77
Total	377	57	68	502

Spearman rank R = 0.741, *p* < 0.001; Pearson chi-square: χ^2^ = 169.231; *p* < 0.001. LRR: Locoregional recurrence.

## Data Availability

The data presented in this study are available on request from the corresponding author.
